# Investigating the motivating factors that influence the adoption of blended learning for teachers’ professional development

**DOI:** 10.1016/j.heliyon.2024.e34900

**Published:** 2024-07-20

**Authors:** Nisar Ahmed Dahri, Noraffandy Yahaya, Waleed Mugahed Al-Rahmi, Haitham Ameen Noman, Fahad Alblehai, Yusri Bin Kamin, Rahim Bux Soomro, Anna Shutaleva, Ahmad Samed Al-Adwan

**Affiliations:** a Faculty of Social Sciences and Humanities, School of Education, University Technology Malaysia, Malaysia; b Computer Engineering, King Abdullah II School of Engineering, Princess Sumaya University for Technology, Khalil Saket Street Al-Jubeiha 11941, P.O. Box 1438, Amman, Jordan; c Computer Science Department, Community College, King Saud University, Riyadh, 11437, Saudi Arabia; d Department of Business Administration, Shah Abdul Latif University, Khairpur, Pakistan; e Ural Federal University Named After the First President of Russia B. N. Yeltsin, 620002, Ekaterinburg, Russia; f Department of Business Technology, Hourani Center for Applied Scientific Research, Business School, Al-Ahliyya Amman University, Amman, Jordan

**Keywords:** Blended learning, Education, Engagement, Technology adoption, Educational environment, Professional development, Developing nations, Teachers

## Abstract

Blended learning (BL), a teaching method merging online and face-to-face learning, is lauded for its potential to enrich educational outcomes and tackle challenges entrenched in conventional teaching practices. In countries like Pakistan, where equitable access to quality professional development remains an obstacle, BL is a promising avenue to surmount training barriers. While BL adoption has evolved swiftly, research into its integration within teacher training remains limited. Notably, no comprehensive model exists describing the motivational factors influencing teachers' perceptions and intentions regarding the blended mode of teacher training. This study aims to identify the motivational elements that motivate schoolteachers in teacher training institutions in Pakistan to incorporate blended learning into their programs. The motivational factors identified in BL literature have been employed to craft a motivation model grounded in their causal relationship. This quantitative study examines the interplay between multiple motivational factors and their impact on BL adoption within teacher training and the BL environment. Surveying 350 schoolteachers (participants) from teacher training institutions, we employed Structural Equation Modeling (SEM) techniques with Smart PLS 4.0 for data analysis. Results reveal that extrinsic and intrinsic motivational factors significantly influence teachers' motivation to adopt BL for training. Notably, "overall training quality" and "educational environment" were non-influential. Overall, the findings underscore that considering a blend of extrinsic and intrinsic factors can wield a 65 % influence on BL adoption. The study's results provide practical guidance for educational leaders, curriculum designers, and faculty members aiming to cultivate a unified blended learning environment for teacher professional development. These insights also underscore the importance of incorporating essential motivational factors into forthcoming blended learning training programs.

## 1 Introduction

Sustainable and equitable access to high-quality education has remained a global challenge [1]. Among the key stakeholders shaping the educational landscape, teachers play a pivotal role in the teaching and learning process [2,3]. Consequently, well-designed professional development programs are crucial in ensuring effective teaching practices and the delivery of exceptional education [4]. Nevertheless, concerns regarding the sustainability of these interventions linger. In-service training remains essential to keeping knowledge, skills, and competencies updated and refreshed. During such training, teachers gain valuable insights into content knowledge, teaching and assessment approaches, and transformative learning experiences, ultimately enhancing their professional competency [[5], [6], [7]]. Numerous studies have demonstrated that professional development interventions enhance teachers' knowledge and abilities, equipping them with a comprehensive and profound understanding of their subjects. In the province of Sindh, traditional Continuous professional development (CPD) models, like the cascade model and top-down training technique, have been used for years. However, these models face significant limitations due to the lack of technology integration and the absence of follow-up assistance [[8], [9], [10], [11]]. At the provincial level, various assessment surveys, such as the SAT 2017–18 (Standardized Achievement Test), reveal that teachers in government schools in Sindh, both at primary and secondary levels, are often underqualified and inadequately trained. They lack essential competency levels in fundamental subjects like languages, mathematics, and science, with limited success [8,12].

The existing teacher development programs lack standardization and sufficient support for teachers' professional improvement [4,6,13,14]. Despite the efforts to improve CPD, Pakistan faces significant challenges in conducting face-to-face teacher training. One of the key challenges is the country's vast geographical spread, particularly in rural and remote areas, making it difficult for teachers to access training centers [6,15]. Limited infrastructure and transportation facilities further exacerbate the issue, leading to a lack of accessibility to face-to-face training venues [[16], [17], [18]]. Moreover, socio-economic factors hinder teachers' participation in face-to-face training. Many teachers in Pakistan belong to low-income communities and may find it financially burdensome to attend distant training programs [19,20]. Additionally, cultural norms and family responsibilities, especially for female teachers, may restrict their mobility and limit their ability to attend face-to-face training [4,7,21]. Furthermore, security concerns in certain regions of Pakistan pose a challenge to organizing large-scale training events. In areas affected by political instability or security issues, conducting face-to-face training becomes risky, affecting the professional development opportunities for teachers in those regions [4,7,21].

Pakistan, as a developing country, recognizes the importance of providing quality professional development opportunities for teachers. Among the professional training offered to them are induction training, promotion-linked training, and continuous professional development (CPD) programs. These training courses aim to equip educators with the necessary knowledge and skills to excel in their roles and adapt to changing educational practices [2,4,6,22]. However, the availability and accessibility of such training opportunities are often limited due to various security, socio-economic, and cultural barriers. To address these issues, the education authorities in Sindh have introduced a blended learning approach for teacher professional development. This novel approach combines online and face-to-face modes, allowing participants to engage in online activities and discussions during the first week and attend face-to-face sessions in the second week at their local cluster-level training centers.

In developed countries, professional development programs for educators have increasingly incorporated blended learning approaches. Research in these contexts suggests blended learning can effectively enhance teachers' skills, promote collaborative learning, and provide personalized learning experiences [23]. The flexibility of blended learning allows teachers to engage in self-paced online modules, participate in virtual discussions, and then apply their knowledge during face-to-face interactions. Such an approach is particularly beneficial for adult learners, allowing them to integrate new knowledge and skills into their pedagogical practices [[23], [24], [25]]. This situation calls for thoroughly examining the teacher development landscape and exploring alternative approaches, such as blended learning, to address these pressing challenges and improve teachers' overall education in Sindh.

Considering the importance of technology usage, the Provincial Institute of Teacher Education (PITE) Sindh has recently introduced a blended learning-based approach for professional development programs for in-service teachers in Sindh to enhance teachers' pedagogical skills [17]. While many professional development programs emphasize technology integration, merely focusing on technology usage skills may not fully leverage the potential of blended learning [26]. Studies have shown that successful blended learning initiatives for teachers integrate pedagogy and content knowledge with technology training, resulting in lasting effects on technology application in the classroom [27]. Engaging teachers in such comprehensive blended learning programs fosters responsiveness for students and leads to more effective adoption of blended learning practices [28]. Pakistan can reap numerous benefits by embracing a BL model for teachers' professional development in the school system. Teachers and students may gain access to online platforms, pre-recorded lectures, interactive instructional models facilitating peer-to-peer and peer-to-faculty interactions, and real-time discussions with experts and experienced individuals in the online space. These advancements promise to enrich the learning experience, promote engagement, and foster greater collaboration within the educational community.

Previous studies have focused on blended learning in general education settings, exploring e-learning content, student achievements, and innovative approaches. However, there is limited research on adopting blended learning from the teacher's training perspective [[26], [27], [28], [29], [30], [31], [32], [33]]. In the digital age, integrating ICT resources has become crucial for managing online and offline activities within the blended learning approach. Despite its potential, blended learning faces challenges related to both students and teachers [34,35]. Teachers require time to adapt to the technology involved in blended learning, and the sudden shift from traditional to blended teaching practices poses another hurdle [26]. Furthermore, the scarcity of in-service teacher training programs tailored to blended learning exacerbates the situation. Suboptimal design and delivery techniques also contribute to the challenges faced in implementing this innovative approach [24,36,37].

In the area of blended learning, various widely recognized models for assessing blended learning acceptance have emerged, including the technology acceptance model (TAM), the unified theory of acceptance and use of technology (UTAUT), social cognitive theory, and the diffusion of Innovations theory [38,39]. These models play a pivotal role in assisting educators, instructional designers, and e-learning technology developers in comprehending how to craft, execute, and assess e-learning systems that align with the expectations and requirements of learners [40]. Numerous research studies have delved into the primary factors influencing individuals' decisions to embrace and engage with online systems. The integration of face-to-face and online instruction, spanning from 15 % to 100 % in-person and 20 %–99 % online, can evolve into a comprehensive online learning experience. Nevertheless, prior research has cast doubts on whether these configurations yield the anticipated benefits of enhancing students' motivation to utilize e-learning systems [[40], [41], [42]].

Additionally, limited internet and technology facilities in students' home areas hinder the seamless adoption of blended learning [34,43]. In-depth research on blended learning by Vaughan et al. [33] highlights various factors impacting its effectiveness, including insufficient teacher training, inadequate technology skills, financial constraints, lack of school vision, and the challenge for teachers in transitioning from content providers to facilitators. These challenges underscore the importance of investigating the use of blended learning in school education in Sindh to understand the factors influencing its adoption within this context. It is essential to explore teachers' intentions and attitudes toward blended learning to inform policy decisions and optimize the effectiveness of training programs. This research study aims to fill this gap by investigating the key factors influencing the adoption of blended learning for the professional development of schoolteachers in Sindh, Pakistan. By examining teachers' intentions, attitudes, and perceptions toward blended learning, this study seeks to contribute to the understanding of how blended learning can be effectively implemented in the context of developing countries. The findings will inform policymakers and education administrators in Sindh and contribute to the broader literature on blended learning adoption within the developing world.

### 1.1 Teachers training and blended learning approach in Sindh

Blended learning for teacher training combines one-to-one technology and face-to-face instruction to deliver content and skills [37]. Various researchers define blended learning as face-to-face and online learning [44]. It is recognized as a combination of traditional classroom practices and online elements [45], with consideration for contextual realities while defining this approach. First, Face-to-face instruction occurs in a physical classroom [46], emphasizing trainer-led lectures/sessions and practical activities [47]. Evaluation in face-to-face learning is typically conducted systematically under teacher supervision, providing participants with quick solutions to queries and hands-on experiences [48]. Trainees' satisfaction with face-to-face learning has been reported to be higher in some studies [49], while others found greater satisfaction with online learning [46]. Secondly, online learning, or web-based learning, delivers instructional content over the Internet, offering flexible access to teaching materials and enabling learning from any location at any time [48]. It utilizes various learning resources, such as presentations, lectures, documents, images, and videos, to create an independent learning environment for students [48]. While online learning presents solutions to traditional education challenges, it also introduces new issues related to remoteness, lack of feedback, and group disconnection [49]. In this context, blended learning has emerged as a promising solution, combining the benefits of both approaches [50].

Blended learning is considered when at least 80 % of the training material is delivered online [44], offering a balanced and integrated approach to address online and face-to-face learning challenges. By leveraging the strengths of both methods, blended learning optimizes teacher training and enhances the learning experience for participants. In response, Sindh, one of the provinces in Pakistan, has introduced a blended learning model of teacher training. The blended learning approach in Sindh typically involves an initial online phase, where participants engage in self-paced activities, followed by a face-to-face phase, which allows for interactive discussions, collaboration, and hands-on learning. This blended learning model aims to address the limitations of traditional face-to-face training and increase participation rates, particularly among female teachers who may face cultural and logistical constraints. While blended learning holds promise in the context of teacher training in Sindh, little research has been conducted to understand the factors influencing its adoption. Studies conducted in developed countries have identified several critical factors influencing blended learning adoption, which need to be explored in the context of Sindh.

### 1.2 Teachers' motivating factors for blended learning adoption

[51] several factors influence teachers' motivation to embrace blended learning (BL), including interest, independent learning, personalized learning, computer self-efficacy, social perception, external expectations, and skill enhancement. These factors are categorized into two main groups: intrinsic and extrinsic influences, based on teachers' experiences with BL practices [44]. Intrinsic factors are rooted in individual teachers’ cognition, such as their instructional philosophy and mental models derived from reflection and experience [52]. External influences, on the other hand, encompass cultural, structural, or instructional elements [53]. factors like perceived usefulness, professional support, technical assistance availability, funding, preparation time, institutional infrastructure, senior staff involvement, and efficacy are pivotal determinants of instructors' motivation to implement BL in higher education institutions. These factors complement the earlier intrinsic and extrinsic factors highlighted by Ref. [44] and collectively shape instructors' motivation to adopt BL approaches.

#### 1.2.1 Intrinsic motivating factors

Teachers' Engagement: One intrinsic motivating factor that significantly impacts the adoption of blended learning is teachers' engagement. Engaged teachers actively participate in the learning process, interact with students, and invest time and effort in developing their teaching skills. This engagement enhances their motivation and creates a more dynamic and supportive learning environment [54]. Active participation and enthusiasm among teachers are crucial drivers of successful blended learning adoption. Teacher Learning: Intrinsic motivation can also stem from a teacher's continuous learning and professional development. Teachers who engage in ongoing learning experiences tend to be more motivated to incorporate innovative teaching methods, such as blended learning, into their pedagogical repertoire. Their commitment to self-improvement and keeping up with educational advancements positively influences their motivation [55]. Blended Learning Motivation: Teachers' motivation to adopt blended learning is an intrinsic factor. When teachers believe in the value and benefits of blended learning, their motivation to integrate it into their teaching practices increases. This self-driven motivation is often rooted in a deep understanding of how blended learning can enhance the learning experience for both teachers and students [44]. A review of existing literature sheds light on these motivating factors and provides insights into their significance in fostering the successful implementation of blended learning in the context of teacher's professional development in Sindh, Pakistan.

#### 1.2.2 Extrinsic motivating factors

Information Quality: Extrinsic factors, such as the quality of information provided in blended learning materials, motivate teachers. High-quality, relevant, up-to-date, and well-organized content can positively influence teachers' motivation to adopt blended learning [44,56]. Access to well-designed learning resources enhances their confidence in the effectiveness of blended learning. Training Quality: The quality of training programs and professional development opportunities is another extrinsic motivating factor. Teachers who perceive training programs as comprehensive, well-structured, and relevant to their teaching needs are more likely to be motivated to explore blended learning [57]. Practical training can equip teachers with the skills and knowledge needed to succeed in a blended learning environment. Teaching Presence: Extrinsic motivation can also arise from the presence and support of colleagues and institutional structures. A supportive teaching presence within an educational institution, including collaborative colleagues and senior staff involvement, can foster teachers’ motivation to engage with blended learning [44]. Collaborative environments and institutional support systems can provide the external encouragement needed for instructors to adopt new teaching approaches. Educational Environment: The overall educational environment within an institution, encompassing factors like infrastructure, technological support, and organizational culture, can serve as a significant extrinsic motivating factor [58]. A well-equipped and supportive educational environment can motivate instructors to explore blended learning methods. Understanding the interplay between these intrinsic and extrinsic motivating factors is essential for educational institutions aiming to promote the successful adoption of blended learning among their teachers. By recognizing and addressing these factors, institutions can create an environment that encourages and sustains instructors' motivation to embrace blended learning, ultimately enhancing the quality of education delivery.

In recent years, the educational field has significantly shifted towards integrating technology into teaching and learning processes. Blended learning, an innovative approach that combines face-to-face instruction with online learning, has emerged as a promising solution to meet the evolving needs of modern education. One of the critical determinants of blended learning adoption is teachers' technology acceptance in this context [59]. conducted a case study in Jordan to investigate EFL (English as a Foreign Language) teachers' technology acceptance in blended learning environments. The findings revealed that teachers' attitudes towards technology significantly influenced their acceptance and usage of blended learning tools and resources. Chen et al. [60] explored university teachers' technology acceptance and usage for blended learning, emphasizing the importance of providing adequate training and support to teachers to enhance their acceptance and integration of technology in the classroom. In the context of university students [44], investigated the factors affecting their acceptance of blended learning using a structural equation modeling approach. This study identified perceived usefulness and ease of use as critical factors influencing students' acceptance of blended learning.

Similarly [61], conducted a systematic review of blended learning in higher education, emphasizing the significance of students' acceptance and satisfaction with the approach in improving learning outcomes. Technology acceptance and blended learning adoption also depend on the perceived effectiveness of the strategy in enhancing teaching and learning experiences. Ghavifekr and Rosdy [62] examined the effectiveness of ICT (Information and Communication Technology) integration in schools, highlighting the importance of technology in creating engaging and interactive learning environments.

Moreover, Kay and Kletskin (2012) evaluated the use of problem-based video podcasts to teach mathematics in higher education, demonstrating the potential of technology-enhanced methods in promoting active learning and teachers' engagement. Teachers' engagement plays a pivotal role in the effectiveness of blended learning [63,64]. Scholars like Picciano (2006) have emphasized the importance of students' active participation and motivation in online and face-to-face components of blended learning. Engaged learners are likelier to take ownership of their learning journey and demonstrate better academic outcomes [65]. Moreover, the role of teachers in blended learning environments is crucial. Bliuc et al. discussed the significance of continuous professional development for instructors, enabling them to integrate technology and pedagogy in their teaching practices effectively. Teachers’ willingness to adopt new instructional strategies and tools directly impacts students' learning experiences [66]. The quality of information and content delivered in blended learning environments also influences students' acceptance and Engagement. Fischer et al. [67] highlighted the importance of providing accurate and up-to-date information in online modules to enhance learners' comprehension and learning outcomes. Additionally, Jaggars and Xu [68] emphasized the significance of instructional design in ensuring the quality of blended learning materials.

The training quality and support provided to students and teachers during the implementation of blended learning are decisive for successful adoption. Adequate training and support systems are essential to address challenges and concerns during the transition to blended learning [5]. Furthermore, the success of blended learning adoption is influenced by the overall educational environment, including elements such as teachers' engagement, teachers learning, information quality, training quality, teaching presence, and educational environment [45]. explored teachers' attitudes and perceptions towards blended learning, emphasizing teaching presence in creating a supportive and engaging learning environment. Teaching presence, defined as the instructor's ability to facilitate and support learning, is another critical factor influencing blended learning adoption. Researchers like Anderson et al. [69] have highlighted the importance of building a strong teaching presence in blended learning environments to foster effective communication and interaction among students and instructors.

[48] They have investigated the adoption of blended learning among engineering students in Malaysia, identifying information quality as a critical factor influencing students' acceptance and satisfaction with the approach. Finally, adopting blended learning has significant implications for educational institutions and their stakeholders. Sarrab and Ayedh [70] examined the impact of blended learning adoption on students' academic performance, revealing positive outcomes associated with this approach. Zhao and Yang [71] systematically reviewed blended learning adoption in higher education, providing insights into the challenges and benefits of this innovative approach.

Furthermore, the educational environment in which blended learning is implemented significantly influences its acceptance and success. Picciano [65] highlighted the need to create a supportive and conducive learning environment that encourages active participation, collaboration, and self-directed learning. As a construct derived from literature, blended learning motivation encompasses students' drive and willingness to engage in blended learning activities. Factors such as the perceived usefulness and ease of use of technology, as proposed by Venkatesh et al. [72], significantly impact students' motivation to adopt blended learning practices.

Finally, the intention to adopt blended learning refers to the readiness of educational institutions and stakeholders to support this innovative approach. Institutional support, infrastructure, and policy frameworks influence the intention to adopt blended learning [73]. This literature review aims to explore the existing research on technology acceptance and its impact on blended learning adoption, highlighting the significance of these factors in shaping the successful implementation of this educational approach. Technology acceptance plays a central role in the successful adoption of blended learning. The literature suggests that teachers’ engagement, teachers learning, information quality, overall training quality, teaching presence, educational environment, blended learning motivation, and intention to adopt blended learning are significant factors that shape the implementation and effectiveness of this educational approach.

## 2 Theoretical background

**Fig. 1:**
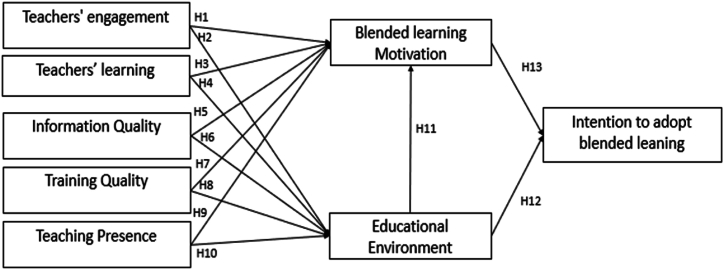
Proposed research hypothesis.

Motivating factors significantly influence teachers' adoption of blended learning (BL). These factors shape an individual teacher's experience when engaging with BL practices for their professional development. The extrinsic determinants and key motivators for adopting BL include information quality, training quality, teaching presence, and the educational environment. In contrast, intrinsic factors, such as teachers' engagement, instructor learning, and BL motivation, assume paramount importance as motivating forces when educators utilize online tools for instructional purposes. The motivation model proposed in Fig. 1 highlights the structured framework wherein the motivation for adopting BL is the dependent variable, while the motivational factors, namely extrinsic and intrinsic, assume the roles of independent variables. On the other facet of this model, the educational environment and the motivation for implementing BL are positioned as the dependent variable, while the adoption of BL is positioned as the independent variable. Fig. 1 also represents the conceptualized motivational model.

The significance of motivating factors in adopting BL is well-documented in educational research. For instance, the Technology Acceptance Model (TAM) proposed by Davis [74] highlights the importance of perceived ease of use and perceived usefulness as key determinants of technology adoption. Extending this model to the educational context, Venkatesh and Bala [75] highlighted that educators' perceptions of the quality and utility of BL platforms significantly influence their willingness to adopt such technologies.

## 3 Hypothesis development

### 3.1 Teachers’ engagement influences BL motivation and educational environment

Teachers’ engagement is a significant factor in the success of blended learning (BL) environments, as it reflects the degree to which teachers are invested in and interact with their professional development activities. Engagement involves interest, attention, participation, and collaboration [76]. Research has consistently shown that higher levels of engagement lead to better outcomes in BL settings.

Lee and Choi (2018) demonstrated that BL positively impacts student engagement by facilitating personalized learning experiences and enhancing interaction with course materials and peers [54]. This engagement translates to increased motivation to participate in BL activities. The personalization and flexibility offered by BL cater to individual learning preferences and needs, thereby fostering a deeper connection to the learning material [54,[77], [78], [79], [80]]. Al-Azawei et al. (2017) found that students engaged in BL settings showed higher motivation and active participation levels, suggesting that engagement is a crucial driver of motivation [81]. Studies like those by Almoslamani [82] and Bond et al. [83] further support the positive correlation between engagement and motivation in BL environments. Nikomo et al. [84] highlighted that engaged students find BL environments motivating due to the increased interaction and feedback mechanisms inherent in such settings. Similarly, Kahu [85] emphasized that engagement fosters a sense of belonging and purpose, enhancing motivation. These findings are echoed in the work of Trowler (2010), who noted that engaged learners exhibit higher levels of intrinsic motivation, which is crucial for sustaining long-term participation in BL activities [86].

Student engagement also significantly contributes to a positive educational environment. Krause and Coates [87] found that higher levels of student engagement are associated with a more supportive and conducive learning environment. Engaged students participate more actively in collaborative learning activities, creating a more dynamic and interactive classroom atmosphere. This active participation helps create a learning environment where students feel supported and valued, essential for effective learning.

Chen and Jang (2010) reported that student engagement leads to a more collaborative and interactive educational environment, benefiting both students and instructors. Their study indicated that engaged students are more likely to engage in meaningful discussions, provide constructive feedback, and support their peers, all of which contribute to a positive educational climate [88]. Additionally, Fredricks et al. (2004) highlighted that engagement, encompassing behavioral, emotional, and cognitive dimensions, plays a pivotal role in creating an environment conducive to learning [89].

Moreover, Skinner and Belmont (1993) found that engaged students foster a more vibrant and interactive educational setting, positively impacting peers and instructors. This supportive environment enhances student learning outcomes and encourages teachers to adopt innovative teaching methods and integrate BL into their instructional practices [90]. Another scholar also focused on student engagement like, Mazer et al. [91] found that student engagement in online discussions and activities significantly enhances their motivation and perception of the course's value. The study by Dabbagh and Kitsantas [92] also indicated that engaged learners are more likely to utilize self-regulation strategies, further boosting their motivation and performance in BL environments.

Reeve [93] also highlighted that engaged students are more proactive in their learning, seeking out additional resources and opportunities to enhance their understanding. This proactive behavior contributes to a more enriching educational environment, encouraging continuous learning and improvement.

The relationships highlight that Engaged teachers are more likely to find BL environments motivating and contribute to a positive and conducive educational setting. This highlights the importance of fostering engagement in BL initiatives to enhance motivation and create a supportive learning environment.

Hypothesis 1 (H1): Teachers' engagement positively influences BL motivation.

Hypothesis 2 (H2): Teachers' engagement positively influences the educational environment.

### 3.2 Teachers’ learning influences BL motivation and educational environment

Teachers' continuous professional development and learning play a pivotal role in successfully adopting and implementing blended learning (BL) environments [15]. When teachers actively engage in learning and skill enhancement, they are better equipped to utilize BL tools and methodologies [17] effectively. This ongoing learning enhances their instructional capabilities and significantly influences their motivation to adopt and integrate BL into their teaching practices [18,64].

Hsu and Chang (2018) demonstrated that teachers who received proper training and support in using technology were more likely to adopt BL in their teaching practices [94]. Their study highlighted the importance of continuous professional development in fostering a positive attitude towards BL [94]. Similarly, Olcott et al. (2015) emphasized that instructors who engage in ongoing learning and training are better equipped to design engaging and interactive BL experiences, enhancing their motivation to use BL tools. These findings suggest continuous learning and professional development are critical for motivating teachers to adopt BL [95].

Al-Fraihat et al. [55] reported that instructors' continuous learning positively influenced student motivation and engagement in BL settings. This indicates that when teachers are motivated and well-prepared through ongoing education, they are more likely to create motivating and engaging BL environments for their students [44]. Moreover, the study by Teo [96] found that teachers' attitudes toward technology and BL are significantly shaped by their professional development experiences. Teachers who participated in comprehensive training programs exhibited higher motivation to integrate BL into their teaching [94,97,98].

Teachers' learning and professional development also significantly impact the educational environment in BL settings. Well-trained and knowledgeable teachers are essential for creating a supportive and conducive learning environment that fosters student engagement and participation [16]. Continuous professional development ensures teachers are current with the latest pedagogical strategies and technological advancements, enabling them to create more effective and interactive BL environments [88,99].

Research by Xie et al. [100] found that high-quality training programs positively affected the learning experiences of both teachers and students, creating a supportive academic environment. Their study highlighted the importance of well-designed training programs in enhancing the overall educational environment in BL settings. Similarly, the survey by Al-Dheleai and Tasir (2020) emphasized that teachers' ongoing learning and development are crucial for fostering a collaborative and interactive educational environment. Engaged and well-trained teachers are likelier to implement innovative teaching methods and create a dynamic learning atmosphere [101].

Hsu et al. [102] also reported that teachers' continuous learning contributes to a positive and supportive educational environment. Their study found that teachers who regularly engage in professional development activities are likelier to adopt student-centered teaching approaches, promoting a more interactive and engaging learning environment. Additionally, the research by Yurdakal et al. [103] indicated that teachers' professional development positively impacts the overall quality of the educational environment, leading to improved student learning outcomes and satisfaction.

Further supporting these hypotheses, Lawless and Pellegrino [104] found that professional development focusing on technology integration significantly enhances teachers' ability to create motivating and effective BL environments. Their study indicated that continuous learning and skill enhancement are critical for teachers to keep pace with technological advancements and pedagogical innovations. The research by Ref. [105] also emphasized the importance of sustained professional development in enabling teachers to design and implement effective BL strategies.

Moreover, Darling-Hammond et al. highlighted that ongoing professional development is essential for teachers to adapt to the evolving educational landscape and meet the diverse needs of their students. Their study found that teachers who participate in continuous learning opportunities to adopt innovative teaching methods and create a positive educational environment that supports student learning and engagement [106].

In a nutshell, the relationships between teachers' learning, BL motivation, and the educational environment is well-supported by existing literature. Continuous professional development and learning motivate teachers to adopt BL and create a supportive and conducive academic environment.

Hypothesis 3 (H3): Teachers' learning positively influences blended learning motivation.

Hypothesis 4 (H4): Teachers' learning positively influences the educational environment.

### 3.3 Information quality influences BL motivation and educational environment

Information quality refers to the content's accuracy, relevance, and credibility within the blended learning (BL) environment [107]. High-quality information is crucial for fostering motivation among teachers and students, as it directly impacts the perceived usefulness and effectiveness of BL tools and resources [108]. When the content is well-designed, clear, and comprehensive, it enhances the learning experience and encourages greater engagement and motivation [109].

Kintu et al. [110] highlighted the importance of providing high-quality information in BL environments to enhance its acceptance and effectiveness. Their study found that the quality of the information provided significantly influenced students' motivation to engage in BL activities. Similarly, Aghbari et al. [111] reported that well-designed and high-quality content in online modules improved overall student motivation by providing clear and comprehensive learning resources. This indicates that the availability of relevant and high-quality information is a critical factor in motivating teachers and students to engage in BL [112].

The study by Al-Fraihat et al. (2020) also emphasized the positive relationship between information quality and student motivation in BL settings. They found that students who perceived the information provided in BL courses as accurate and relevant were more motivated to participate in learning activities [113]. Furthermore, the research by Stumbrienė el al [114]. indicated that high-quality information positively influences teachers' motivation to adopt BL, ensuring that the content they deliver is effective and valuable to their students.

High-quality information also plays a crucial role in shaping the educational environment in BL settings. Accurate and relevant content enhances learning outcomes and contributes to creating a supportive and conducive learning environment [115]. When teachers and students have access to reliable and comprehensive information, it fosters a positive educational atmosphere that promotes engagement, collaboration, and active participation [110].

Law et al. [116] found that the overall quality of information provided in BL environments significantly impacts the educational environment. Their study highlighted that high-quality content improves the learning experiences of both teachers and students, creating a more supportive and interactive educational setting. Similarly, Dahri et al. [17] emphasized the importance of information quality in fostering a positive academic environment, as it ensures that students have access to the necessary resources to succeed in their studies.

Research by Tsai el al [117]. also indicated that information quality is a key determinant of the educational environment in BL settings. They found that students who perceived the information as credible and relevant were likelier to engage in collaborative and interactive learning activities, enhancing the overall educational environment. Idkhan et al. [118] found that information quality significantly influences the success of e-learning environments by enhancing user satisfaction and engagement. Their study indicated that high-quality content is essential for creating a motivating and effective learning experience. The research by Refs. [119,120] also emphasized the importance of information quality in shaping the educational environment, as it directly impacts user satisfaction and perceived usefulness.

These relationships between information quality, BL motivation, and the educational environment are found to be highly significant. High-quality information is essential for motivating teachers and students to engage in BL and creating a supportive and conducive academic environment. This underscores the importance of providing accurate, relevant, and credible content in BL settings to enhance learning outcomes and foster a positive educational atmosphere.

Hypothesis 5 (H5): Information quality positively influences blended learning motivation.

Hypothesis 6 (H6): Information quality positively influences the educational environment.

### 3.4 Training quality influences BL motivation and educational environment

Training quality refers to the design, delivery, and effectiveness of training programs provided to both teachers and students in blended learning (BL) environments [[121], [122], [123]]. High-quality training is essential for equipping teachers with the necessary skills and knowledge to effectively utilize BL tools and resources, which in turn enhances their motivation to adopt and integrate BL into their teaching practices [15,18].

Numerous studies have highlighted the importance of training quality in influencing teachers' motivation to engage in BL. For instance, Porter et al. [124] demonstrated that instructors who received proper training and support in using technology were more likely to adopt BL in their teaching practices. Their study indicated that well-designed training programs that effectively convey the benefits and usage of BL tools significantly boost teachers' confidence and willingness to integrate BL into their curricula.

Similarly, various studies [125,126] emphasized that ongoing professional development and continuous learning opportunities are crucial for maintaining high motivation levels among teachers. They found that instructors who participated in regular training sessions were better equipped to design engaging and interactive BL experiences, which enhanced their motivation and positively influenced their students' motivation to participate in BL activities.

Moreover, Wang et al. [127] indicated that high-quality training programs are vital for sustaining teachers' motivation to use BL. Their research showed that when teachers perceive the training they receive as comprehensive, relevant, and effective, they are more likely to feel motivated to adopt BL. This underscores the importance of providing continuous, high-quality training to ensure teachers remain motivated to integrate BL into their teaching practices.

High-quality training programs also play a critical role in shaping the educational environment in BL settings [57]. Effective training ensures that teachers and students are well-prepared to navigate and utilize BL tools, which creates a supportive and conducive educational environment [128]. When well-trained, teachers can design and deliver high-quality BL experiences that foster student engagement, collaboration, and active participation.

Yang and Jie [128] found that the overall quality of training provided in BL environments significantly impacts the educational environment. Their study highlighted that high-quality training improves the learning experiences of both teachers and students, creating a more supportive and interactive educational setting. Similarly [129], emphasized the importance of training quality in fostering a positive academic environment, ensuring that teachers and students have the necessary skills and knowledge to succeed in their BL activities.

Research by García et al. [130], also indicated that training quality is a key determinant of the educational environment in BL settings. They found that students who received effective training were more likely to engage in collaborative and interactive learning activities, thereby enhancing the overall educational environment. Further, Liu et al. [131] highlighted that high-quality training programs foster student engagement and participation in the academic environment. Their study indicated that when students receive comprehensive training on using BL tools, they are more likely to feel confident and motivated to engage in BL activities.

The relationships highlight that High-quality training is essential for motivating teachers and students to engage in BL and creating a supportive and conducive educational environment [132]. This underscores the importance of providing comprehensive, relevant, and effective training programs in BL settings to enhance learning outcomes and foster a positive educational atmosphere.

Hypothesis 7 (H7): Training quality positively influences blended learning motivation.

Hypothesis 8 (H8): Training quality positively influences the educational environment.

### 3.5 Teaching presence influences educational environment and blended learning motivation

Teaching presence, encompassing instructor engagement, communication, feedback, and support within the blended learning (BL) environment, is a critical factor influencing student motivation [48,133]. Effective teaching presence ensures that students feel supported, guided, and connected to their instructors, enhancing their motivation to participate in BL activities [45].

Numerous studies have highlighted the importance of teaching presence in influencing student motivation in BL environments [44,45]. For instance, Villanueva [134] found that a strong teaching presence, characterized by clear communication and active instructor involvement, significantly increased students' motivation to engage in BL courses. Their study demonstrated that when students perceive their instructors as accessible and responsive, they are more likely to feel motivated to participate actively in BL activities.

Similarly, the research by Garrison, Olson et al. (2017) emphasized that teaching presence is a crucial determinant of students' motivation in BL settings. They found that instructors facilitating discussions effectively, providing timely feedback, and creating a supportive learning environment significantly enhance students' intrinsic motivation to learn [135]. This is supported by the findings of Almasi, Mustapha, and Chang Zhu.(2023), who reported that students' perception of teaching presence positively influences their motivation and engagement in BL courses [136].

Moreover, the study by Almulla [137] indicated that teaching presence plays a vital role in sustaining students' motivation in BL environments. They found that when instructors demonstrate a strong teaching presence, students are more likely to feel connected to the course content and motivated to participate actively. This underscores the importance of maintaining high levels of teaching presence to ensure students remain motivated throughout their BL experience.

Teaching presence also significantly contributes to shaping the educational environment in BL settings. Effective teaching presence creates a sense of community, fosters collaboration, and enhances the overall learning experience, thereby contributing to a positive and conducive educational environment [138].

Research by Shea et al. [139] highlighted the impact of teaching presence on the educational environment in BL courses. They found that a strong teaching presence, characterized by active instructor engagement and effective communication, creates a supportive and interactive learning atmosphere. This study emphasized that when instructors actively facilitate discussions and provide feedback, it enhances the overall educational environment, making it more conducive to learning.

Similarly, the study by Arbaugh [140] indicated that teaching presence is a critical factor in creating a positive educational environment in BL settings. They found that when instructors demonstrate a strong teaching presence, it fosters a sense of community and collaboration among students, thereby enhancing the overall learning experience. Another study reported that teaching presence positively influences the educational environment by promoting student engagement and interaction [141].

These relationships highlight that Effective teaching presence is essential for motivating students to engage in BL activities and for creating a positive and conducive educational environment. This underscores the importance of maintaining high levels of teaching presence to enhance learning outcomes and foster a supportive educational atmosphere in BL settings.

Hypothesis 9 (H9): Teaching presence positively influences blended learning motivation.

Hypothesis 10 (H10): Teaching presence positively influences the educational environment.

### 3.6 Educational environment influences blended learning motivation and intention to adopt blended learning

The blended learning (BL) educational environment encompasses various factors, including institutional support, technological infrastructure, collaborative learning opportunities, and overall classroom dynamics [115,117]. A supportive and well-structured educational environment enhances students' motivation to engage in BL activities [58].

Numerous studies have underscored the importance of a positive educational environment in influencing BL motivation [44,48]. For instance, Al-Osaimi, Dalyal Nader, and Mirna Fawaz [142] found that a well-designed educational environment with adequate technological support and resources significantly enhances students' motivation to participate in BL. Their study demonstrated that when students perceive their learning environment as supportive and conducive to learning, they are more likely to feel motivated to engage in BL activities.

Similarly, the research by Goh, Tiong-Thye, and Bing Yang [143] emphasized the role of the educational environment in fostering BL motivation. They found that a positive educational environment, characterized by collaborative learning opportunities and effective communication channels, significantly boosts students' motivation to participate in BL. This is supported by the findings of Martin and Bolliger (2021), who reported that students' perception of a supportive and interactive educational environment positively influences their motivation and engagement in BL courses [143].

The educational environment also significantly influences the intention to adopt BL among educators and institutions [144]. A supportive educational environment fosters a positive attitude toward BL adoption by providing the necessary resources, training, and institutional support. Research by Porter, Graham, Spring, and Welch [145] highlighted the impact of the educational environment on the intention to adopt BL. They found that institutions with a supportive educational environment characterized by strong leadership, adequate resources, and professional development opportunities are likelier to adopt BL practices. This study emphasized that when educators perceive their environment as supportive and conducive to BL, they are more likely to adopt and implement BL in their teaching practices.

Similarly, various studies indicated that the educational environment is a critical determinant of BL adoption intention. They found that when institutions provide adequate technological infrastructure and support, it significantly influences educators' intention to adopt BL [[145], [146], [147]].

This literature highlights that a positive and supportive educational environment is essential for motivating students to engage in BL activities and encouraging educators to adopt and implement BL practices. This evident the importance of creating a conducive educational environment to enhance learning outcomes and foster the adoption of BL in educational institutions.

Hypothesis 11 (H11): The educational environment positively influences blended learning motivation.

Hypothesis 12 (H12): The educational environment positively influences the intention to adopt blended learning.

### 3.7 Blended learning motivation influences the intention to adopt blended learning

Blended learning (BL) motivation refers to the willingness and enthusiasm of educators and students to engage in blended learning environments [44]. This motivation is often driven by perceived benefits such as enhanced learning experiences, increased flexibility, and improved teaching effectiveness. Understanding the factors that motivate teachers to adopt BL is crucial for promoting its widespread implementation [148,149].

Studies have shown a strong link between BL motivation and the intention to adopt BL. When educators are motivated to engage in BL [[150], [151], [152]], they are more likely to integrate these practices into their teaching methodologies. For instance, the study by Yu et al. [153] found that teachers' motivation to adopt BL was significantly influenced by their perceptions of its benefits, such as improved student engagement and learning outcomes. The researchers emphasized that motivated teachers are more likely to adopt innovative teaching practices, including BL, to enhance their professional development and teaching effectiveness.

Similarly, the research by Refs. [148,154] highlighted that teachers' motivation to adopt BL is often driven by the desire to improve instructional practices and student learning experiences. They found that educators who recognize the advantages of BL, such as personalized learning and greater student interaction, are more inclined to adopt these practices in their teaching. This suggests that enhancing teachers' motivation to adopt BL can lead to a higher likelihood of its implementation in educational settings.

Moreover, the study by Wang and Tianchong [155] emphasized that teachers' motivation to adopt BL is often linked to their professional development needs and the desire for continuous improvement. The researchers found that educators motivated to enhance their teaching skills and stay updated with the latest educational technologies are more likely to adopt BL. This suggests that professional development programs focusing on BL can significantly enhance teachers' motivation to adopt these practices.

In addition to the studies mentioned above, several other research works have explored the relationship between BL motivation and the intention to adopt BL. For example, the study by Moskal, Dziuban, and Hartman [156] found that motivated educators, who recognize the benefits of BL for student engagement and learning outcomes, are more likely to adopt these practices. Their research emphasized the importance of highlighting the advantages of BL to enhance teachers' motivation and promote its adoption.

Similarly, the research by Refs. [44,155,157] indicated that educators' motivation to adopt BL is influenced by their perceptions of its effectiveness and the support provided by their institutions. The study found that motivated teachers who perceive BL as an effective teaching method and receive adequate institutional support are more likely to adopt these practices.

BL motivation and the intention to adopt BL Motivated educators who recognize the benefits of BL and feel confident in their ability to implement these practices are more likely to adopt BL in their teaching. This underscores the importance of enhancing teachers' motivation to promote the widespread adoption of BL in educational institutions. By providing professional development opportunities and highlighting the advantages of BL, institutions can foster a supportive environment that encourages educators to embrace these innovative teaching practices.

Hypothesis 13 (H13): Blended learning motivation positively influences the intention to adopt blended learning.

## 4 Research methodology

### 4.1 Sample and data collection

A convenience sampling technique was employed to select a representative sample of 350 participants. The sample consisted of teachers from various schools and educational institutions in the Sindh province. Participants were selected based on their involvement in the blended learning mode of training and willingness to participate in the study. Data collection was carried out through a structured questionnaire survey. The questionnaire consisted of two sections: demographic information and the Likert-scale items measuring the constructs of interest of the study (See questionnaire in Appendix A). The questionnaire was distributed electronically to the participants, and they were given a designated timeframe to complete and submit their responses. The confidentiality and anonymity of the participants were ensured during the data collection process.

### 4.2 Online training through MS Teams platforms

The online training mode of teachers: in this innovative two-week teacher training program, educators are immersed in a dynamic learning experience using Microsoft Teams, fostering collaboration, critical thinking, creativity, and effective teaching methodologies. The program's structure involves hosting the entire training on Microsoft Teams, providing participants access to daily tasks, reading materials, quizzes, group activities, and assignments. Each day features a 3-h live online training session, where participants engage in real-time discussions with trainers and peers. The platform offers various features, including virtual meetings for interactive talks, meticulous task planning, easy access to training materials, quizzes for self-assessment, breakout rooms for collaborative group work, simplified assignment submission, and visual progress tracking. These elements combine to stimulate creativity and critical thinking among participants while enhancing their teaching skills.

### 4.3 Instrument development methodology

The questionnaire utilized in this study was developed based on established scales from the existing literature, primarily drawing from technology acceptance models and related studies on blended learning. The items were adapted to suit the specific context of teachers' professional development in school education. A rigorous instrument development process was followed to ensure the validity and reliability of the questionnaire. The initial draft of the questionnaire was reviewed by a group of experts consisting of experienced researchers and practitioners in education and technology-enhanced learning. Their feedback was incorporated to enhance the items' clarity, relevance, and comprehensibility. The finalized questionnaire underwent a pilot testing phase with a small group of participants (n = 30) not included in the primary sample. The pilot data were analyzed using item and exploratory factor analysis (EFA) techniques to assess the constructs' internal consistency and factor structure [158].

### 4.4 Structural equation modeling (SEM) method

This study employed structural equation modeling (SEM) as the statistical method to analyze and test the hypotheses proposed in the theoretical model [159,160]. SEM offers a powerful approach to exploring the direct impact and causal relationships between multiple variables. The analysis involved two essential steps: 1) Measurement Model analysis [161] and 2) Structural model analysis [162]. The Measurement Model analysis was used to assess the validity and reliability of the measurement scales used in the study, ensuring that the observed variables accurately represent the underlying constructs. Subsequently, the Structural model analysis was conducted to examine the relationships between the constructs and test the hypothesized direct effects. Through these comprehensive SEM analyses, we gain valuable insights into the factors influencing blended learning adoption and their interconnections, allowing us to draw robust conclusions from the data [161].

## 5 Findings

### 5.1 Data analysis and results

The data collected for this study were analyzed using Smart PLS 4.0, a reliable software tool commonly used to assess measurement models in many studies [163,164,165]. The data analysis process involved two distinct levels, encompassing the evaluation of the measurement and structural models, as Hair et al. [166] recommended. The measurement model evaluation focused on validating the measurement scales used in the study, ensuring their reliability and validity in representing the underlying constructs. On the other hand, the structural model evaluation aimed to examine the relationships and direct effects between the constructs, providing valuable insights into the factors influencing the arrangement under investigation. By employing Smart PLS 4.0 and following the guidelines proposed by Hair et al. [167], we ensured a rigorous and robust data analysis, enhancing the credibility and accuracy of our research findings.

### 5.2 Descriptive analysis

The demographic data collected from schoolteachers and officers provide valuable insights into the characteristics and preferences of the participants, as highlighted in Table 2. The age distribution is diverse, with the highest number of participants falling from 31 to 40 (37.2 %), followed by those aged 20 to 30 (27.1 %). Participants aged between 41 and 50 and 51 to 60 comprise 22.9 % and 12.9 % of the sample, respectively. This diverse age distribution indicates that the study encompasses participants from different career stages, offering a comprehensive view of the education workforce in the region. Regarding gender, the data reveals a slightly higher representation of female participants (52.9 %) than male participants (45.7 %). Educational qualification data demonstrates that most participants hold a master's degree (65.7 %), while 22.9 % have a bachelor's degree. A smaller percentage possess a Ph.D./Doctorate (10.0 %), and only 1.4 % have other qualifications. Teaching experience analysis shows a diverse range of experience levels among the participants. Most participants have 6–10 years of experience (25.7 %), and those with 11–15 years of experience constitute 22.9 % of the sample. Teachers with 0–5 years of experience account for 20.0 %, 17.1 % have 16–20 years of experience, and 14.3 % have more than 20 years of experience. The position/designation data indicate that most participants are participants (71.4 %), while the remaining 28.6 % hold higher positions such as Officer, Principal, or Headmaster.

**Table 1:** Constructs, Cronbach's alpha values, and references.

Construct	No of Items	Cronbach's Alpha	References
Teachers' Engagement	4	0.85	[138]
Teachers Learning	4	0.87	[44]
Information Quality	4	0.83	[46,101]
Training Quality	5	0.88	[132]
Teaching Presence	4	0.84	[50], [95]
Educational Environment	4	0.81	[44]
Blended Learning Motivation	3	0.89	[44]
Intention to Adopt Blended Learning	3	0.86	[44,46].

Note: Cronbach's alpha values indicate the internal consistency reliability of each construct. References provide the sources from which the items and scales were adapted. The reliability of the constructs was assessed using Cronbach's alpha coefficient, and Table 1 shows values above 0.70, which indicates good internal consistency [168].

**Table 2:** Demographics information of participants.

Demographic Characteristic	Count	Percentage (%)
Age (years)		
−20 to 30	95	27.1
−31 to 40	130	37.2
−41 to 50	80	22.9
−51 to 60	45	12.9
Gender		
- Male	160	45.7
- Female	185	52.9
Educational Qualification		
- Bachelor's degree	80	22.9
- Master's degree	230	65.7
- PhD/Doctorate	35	10.0
- Other	5	1.4
Teaching Experience (Years)		
−0 to 5	70	20.0
−6 to 10	90	25.7
−11 to 15	80	22.9
−16 to 20	60	17.1
- Above 20	50	14.3
Position/Designation		
- Teacher	250	71.4
- Officer/Principal/HM	100	28.6
Type of School/Institution		
- Public School	300	100.0
Geographic Location		
- Urban	190	77.1
- Rural	80	22.9
Training Experience		
- Yes	300	85.7
- No	50	14.3
Access to Technology		
- Yes	330	94.3
- No	20	5.7
Preferred Mode of Learning		
- Face-to-face	90	25.7
- Online	120	34.3
- Blended	130	37.1
- No preference	10	2.9
Personal Challenges		
- Lack of time	100	28.6
- Lack of resources	60	17.1
- None	160	45.7
- Other	30	8.6
Availability for Training		
- Very willing	120	34.3
- Somewhat willing	150	42.9
- Neutral	50	14.3
- Somewhat unwilling	20	5.7
- Very unwilling	10	2.9

All participants are from public schools, accounting for 100.0 % of the sample. Geographic location analysis reveals that a significant proportion of the participants come from urban areas (77.1 %), with the remaining 22.9 % of rural regions. Regarding training experience and access to technology, the study shows that an overwhelming majority of teachers have prior training experience (85.7 %) and access to technology (94.3 %). The analysis of preferred learning modes demonstrates that blended learning is the most favored choice (37.1 %), followed closely by online learning (34.3 %), while face-to-face learning is preferred by 25.7 % of the participants. A small percentage (2.9 %) expresses no specific preference. The study also considers the personal challenges faced by participants. The most common challenges reported are "lack of time" (28.6 %) and "lack of resources" (17.1 %). Additionally, a significant percentage of teachers (45.7 %) reported facing no personal challenges, while 8.6 % reported other challenges. Finally, the availability for training among participants is gauged, with 34.3 % expressing a "very willing" attitude and 42.9 % being "somewhat willing" to participate in training. A smaller percentage is either neutral (14.3 %), "somewhat unwilling" (5.7 %), or "very unwilling" (2.9 %) to attend training.

As mentioned earlier, structural equation modeling (SEM) operates on two distinct levels, with the first level being the Measurement Model (MM). The MM aimed to assess the loadings of observed variables, i.e., items used to measure latent variables (constructs/factors). Confirmatory Factor Analysis (CFA) techniques were applied to validate the MM, and it was evaluated based on reliability, discriminant validity, and convergent validity, following the guidelines proposed by Hair et al. [167]; the results of this analysis can be found in Table 3, Table 4. Table 3 presents the factor loadings of individual items, and it is worth noting that all factor loadings were above the suggested threshold value of 0.7 [169,170], indicating a strong relationship between the items and their respective constructs. However, it is essential to highlight that OTQ1 and INL4 items had low factor loadings below the threshold values and were consequently removed from further analysis to ensure the robustness of the model. Table 3 also shows that the mean scores for each construct ranged from 3.411 to 4.626, indicating generally positive perceptions across the evaluated factors. The standard deviations ranged from 0.632 to 1.099, suggesting moderate to high variability in participants' responses. Higher mean scores reflected stronger agreement with positive statements about each construct, indicating favorable attitudes towards blended learning and its potential for enhancing professional development. These findings offer valuable information about participants' perceptions and form the foundation for further analysis using structural equation modeling (SEM) to examine the relationships and causalities between the constructs, validating the proposed theoretical model and hypotheses.

**Table 3:** The results of the descriptive statistical analysis of each variable.

Measurement items		Factor Loadings	Mean	SD
**Teachers' Engagement (ENG1-ENG4)**				
ENG1	I was motivated to participate actively in the blended learning training.	0.765	4.063	1.026
ENG2	The training content and activities captured my interest and attention.	0.812	3.866	1.043
ENG3	I felt engaged and involved in the learning process throughout the training.	0.815	3.837	0.985
ENG4	Overall, I found the training experience enjoyable and fulfilling.	0.686	3.803	1.055
**Teachers Learning (INL1-INL4)**				
INL1	The teachers who facilitated the blended learning training were knowledgeable and competent.	0.932	4.383	0.75
INL2	The teachers were responsive to my questions and concerns during the training.	0.951	4.360	0.776
INL3	The teachers effectively explained complex concepts and clarified doubts.	0.935	4.349	0.792
**Information Quality (INQ1-INQ4)**				
INQ1	The information provided in the online learning materials was relevant and current.	0.788	4.609	0.72
INQ2	I found it easy to access and understand the training resources and materials.	0.833	4.603	0.65
INQ3	The training content was well-organized and logically presented.	0.808	4.626	0.632
INQ4	The information provided helped achieve the learning objectives.	0.698	4.403	0.718
**Training Quality (OTQ1-OTQ5)**				
OTQ2	The training objectives and outcomes were communicated at the beginning.	0.649	4.377	0.745
OTQ3	The training met my expectations and learning needs.	0.747	4.326	0.746
OTQ4	I found the blended learning approach to be effective for my professional development.	0.757	4.354	0.721
OTQ5	I would recommend this training to my colleagues and peers.	0.695	4.329	0.743
**Teaching Presence (TPR1-TPR4)**				
TPR1	The teachers provided timely feedback and support during the online learning phase.	0.852	4.360	0.787
TPR2	The teachers actively guided and facilitated face-to-face sessions.	0.909	4.326	0.819
TPR3	The teachers encouraged open communication and discussion among participants.	0.913	4.389	0.791
TPR4	The teachers fostered a positive and inclusive learning environment.	0.875	4.369	0.785
**Educational Environment (EEN1-EEN3)**				
EEN1	The overall learning environment was conducive to my learning experience.	0.872	4.360	0.783
EEN2	The training resources and facilities were accessible and user-friendly.	0.874	4.389	0.747
EEN3	The educational environment fostered a positive and collaborative learning culture.	0.862	4.377	0.749
EEN4	I felt comfortable and supported in the learning environment.	0.803	4.303	0.76
**Blended Learning Motivation (BLN1-BLM3)**				
BLM1	I was motivated to engage in the blended learning training actively.	0.801	3.703	1.01
BLM2	The blended learning approach enhanced my motivation to learn.	0.834	3.580	0.99
BLM3	I believe that blended learning is an effective way to enhance professional development.	0.822	3.626	0.956
**Intention to Adopt Blended Learning**				
IBL1	I intend to adopt blended learning for future professional development.	0.81	3.526	1.024
IBL2	I am likely to recommend blended learning to my colleagues for their training.	0.836	3.411	1.099
IBL3	I plan to incorporate blended learning strategies in my teaching practices.	0.742	3.546	1.062

**Table 4:** Analysis of the reliability and validity of the measurement model.

Construct	Cronbach's alpha	Composite reliability (CR)	Average variance extracted (AVE)
BLM	0.756	0.86	0.671
EEN	0.875	0.914	0.728
ENG	0.774	0.854	0.595
IBL	0.717	0.839	0.635
INL	0.933	0.957	0.882
INQ	0.791	0.864	0.614
OTQ	0.712	0.805	0.509
TPR	0.91	0.937	0.788

As presented in Table 4. The reliability analysis of the measurement constructs revealed internal solid consistency, as evident from Cronbach's alpha values ranging from 0.756 to 0.933 [167,169], surpassing the recommended threshold of 0.7, as suggested by previous studies [167,169]. This indicates that the measurement items within each construct are highly reliable in assessing the targeted constructs. Furthermore, the Composite Reliability (CR) values ranging from 0.805 to 0.957 exceeded the recommended cutoff of 0.7 [170,171], providing additional evidence of the constructs' internal consistency and reliability. To assess the convergent validity of the measurements, we examined the Average Variance Extracted (AVE) values, which ranged from 0.509 to 0.882. All constructs surpassed the suggested threshold of 0.5, as recommended by Refs. [161,169], indicating satisfactory convergent validity.

The results of discriminant validity are presented in Table 5. To ensure discriminant validity, cross-loadings, the Heterotrait Monotrait ratio (HTMT), and the Fornell-Larker criterion were employed [22,169]. Table 5 displays the HTMT ratio results, which demonstrate that the values for each construct do not exceed the threshold of 0.85, confirming the discriminant validity [172].

**Table 5:** Discriminant Validity (HTMT ratio).

	BLM	EEN	ENG	IBL	INL	INQ	OTQ	TPR
BLM								
EEN	0.1							
ENG	0.825	0.137						
IBL	0.973	0.122	0.733					
INL	0.054	0.45	0.066	0.079				
INQ	0.134	0.422	0.11	0.105	0.44			
OTQ	0.054	0.343	0.119	0.043	0.329	0.637		
TPR	0.056	0.433	0.081	0.054	0.318	0.388	0.305	

The Fornell-Larker criteria outcomes are reported in Table 6, indicating that the square roots of the average variance extracted for each construct were higher than the correlations between the constructs [17,164,169,173], further validating the discriminant validity.

**Table 6:** Discriminant Validity (Fornell-Larker criterion).

	BLM	EEN	ENG	IBL	INL	INQ	OTQ	TPR
BLM	**0.819**							
EEN	−0.07	**0.853**						
ENG	0.648	−0.114	**0.771**					
IBL	0.737	−0.09	0.539	**0.797**				
INL	−0.034	0.41	−0.047	−0.051	**0.939**			
INQ	−0.056	0.368	−0.019	−0.051	0.393	**0.784**		
OTQ	−0.011	0.315	0.059	−0.01	0.336	0.545	**0.713**	
TPR	0.001	0.39	−0.041	−0.022	0.294	0.333	0.26	**0.888**

Additionally, cross-loading criteria can be found in Table 7. This analysis determines how well an item aligns with its intended construct compared to other constructs [163]. To ensure discriminant validity, an item should exhibit higher factor loadings with its designated construct and lower cross-loadings with different constructs. In our study, most items demonstrated more substantial factor loadings with their intended constructs, supporting their discriminant validity. The measurement model showed satisfactory reliability and validity, as no significant errors were observed in its development.

**Table 7:** Cross loadings factors.

	BLM	EEN	ENG	IBL	INL	INQ	OTQ	TPR
BLM1	**0.801**	−0.06	0.466	0.526	0.003	−0.08	−0.038	−0.041
BLM2	**0.834**	−0.093	0.569	0.646	−0.083	−0.094	0.001	−0.004
BLM3	**0.822**	−0.019	0.548	0.629	0.003	0.033	0.005	0.041
EEN1	−0.09	**0.872**	−0.105	−0.106	0.386	0.375	0.283	0.32
EEN2	−0.093	**0.874**	−0.117	−0.087	0.338	0.327	0.305	0.34
EEN3	−0.032	**0.862**	−0.107	−0.045	0.361	0.314	0.259	0.362
EEN4	−0.015	**0.803**	−0.051	−0.065	0.307	0.223	0.219	0.308
ENG1	0.487	−0.112	**0.765**	0.278	−0.014	−0.061	0.01	−0.081
ENG2	0.557	−0.128	**0.812**	0.466	−0.082	−0.07	0.013	−0.073
ENG3	0.561	−0.063	**0.815**	0.494	0.002	0.088	0.103	0.039
ENG4	0.355	−0.032	**0.686**	0.424	−0.058	−0.022	0.059	−0.008
IBL1	0.646	−0.139	0.519	**0.81**	−0.079	−0.118	−0.008	−0.031
IBL2	0.639	−0.033	0.344	**0.836**	0.013	0.033	−0.005	0.016
IBL3	0.442	−0.031	0.432	**0.742**	−0.063	−0.034	−0.014	−0.046
INL1	−0.025	0.389	−0.064	−0.056	**0.932**	0.347	0.318	0.28
INL2	−0.032	0.407	−0.034	−0.057	**0.951**	0.384	0.31	0.253
INL3	−0.04	0.355	−0.033	−0.03	**0.935**	0.376	0.322	0.3
INQ1	−0.1	0.229	−0.071	−0.075	0.272	**0.788**	0.383	0.271
INQ2	−0.055	0.294	0.015	−0.046	0.221	**0.833**	0.386	0.206
INQ3	−0.067	0.244	−0.005	−0.054	0.249	**0.808**	0.339	0.292
INQ4	0.023	0.351	−0.008	0	0.443	**0.698**	0.546	0.272
OTQ2	−0.01	0.31	0.019	−0.018	0.409	0.489	**0.649**	0.185
OTQ3	−0.029	0.164	0.032	−0.031	0.106	0.384	**0.747**	0.173
OTQ4	−0.01	0.176	0.054	0.009	0.158	0.285	**0.757**	0.211
OTQ5	0.023	0.15	0.082	0.024	0.113	0.274	**0.695**	0.155
TPR1	0.007	0.315	−0.017	−0.043	0.26	0.252	0.243	**0.852**
TPR2	0.039	0.395	−0.017	0.006	0.312	0.331	0.254	**0.909**
TPR3	−0.027	0.353	−0.06	−0.029	0.23	0.307	0.231	**0.913**
TPR4	−0.021	0.309	−0.054	−0.019	0.234	0.285	0.191	**0.875**

The R2 values are presented in Table 8 represent the coefficient of determination in regression analysis [159], indicating the proportion of variance in the dependent variable explained by the independent variables. Higher R2 values indicate a better fit of the regression model to the data. In this study, the R2 values for blended learning motivation (BLM) and intention to adopt blended learning (IBL) are 0.426 and 0.645, respectively, suggesting that the independent variables can explain 42.6 % and 64.5 % of the variation in BLM and IBL. The R2 values demonstrate varying degrees of model fit for the constructs under investigation, with BLM and IBL showing moderate model fit [159]. Similarly, Salloum and colleagues (2019) argued that an R2 value surpassing 0.10 is acceptable.

**Table 8:** Fitting coefficient of the structural model.

Constructs	R Square
**BLM**	**0.426**
**EEN**	**0.388**
ENG	0.000
**IBL**	**0.645**
INL	0.000
INQ	0.000
OTQ	0.000
TPR	0.000

### 5.3 Structural model analysis

**Fig. 2:**
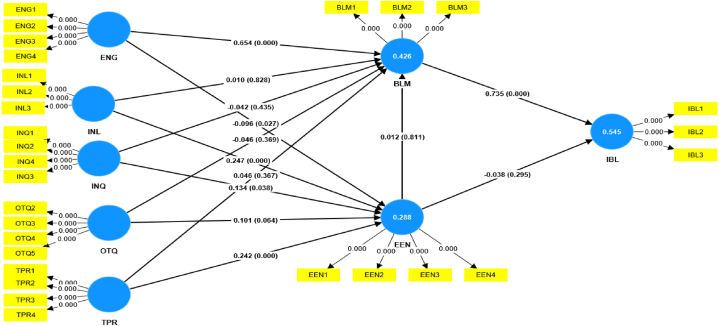
Path coefficients.

**Fig. 3:**
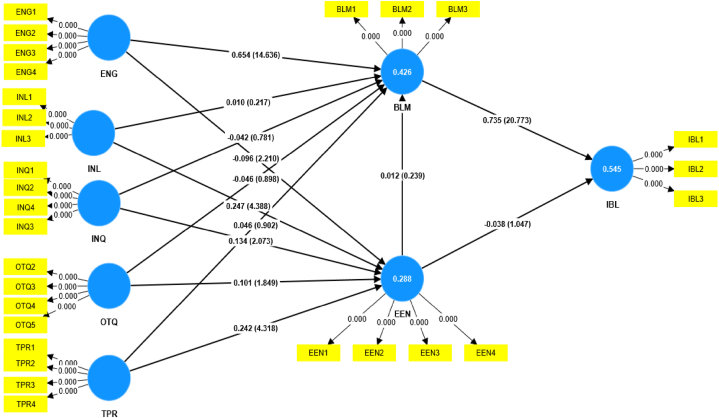
Path (T-Values).

Lastly, in the context of partial least squares (PLS), the bootstrapping technique was applied, using 5000 samples, to assess the significance level of the paths, as indicated by the t-values. For a two-tailed test, these t-values should exceed 1.96, a threshold previously utilized by Ref. [164]. As a result, the findings presented in Table 9, containing path coefficients (β), t-values, and p-values, are utilized to corroborate the hypotheses (H1–H13). Table 9 provides the results of hypothesis testing for the paths in the research model. To validate the research hypotheses and examine the relationships between constructs, SmartPLS 4.0 was utilized. The hypotheses are presented in Table 9, the path coefficient findings are illustrated in Fig. 2, and the corresponding T-values are presented in Fig. 3. These analyses offer valuable insights into the strength and significance of the relationships between the constructs and support the investigation of the key factors influencing the adoption of blended learning for the professional development of schoolteachers in Sindh, Pakistan.

**Table 9:** Hypothesis testing (Path, T-Value, and *P*-value).

Hypothesis	Path Coefficient	T Value	P	Results
BLM - > IBL	0.735	20.773	0.000	Supported
EEN - > BLM	0.012	0.239	0.811	Not Supported
EEN - > IBL	−0.038	1.047	0.295	Not Supported
ENG - > BLM	0.654	14.636	0.000	Supported
ENG - > EEN	−0.096	2.210	0.027	Supported
INL - > BLM	0.01	0.217	0.828	Not Supported
INL - > EEN	0.247	4.388	0.000	Supported
INQ - > BLM	−0.042	0.781	0.435	Not Supported
INQ - > EEN	0.134	2.073	0.038	Supported
OTQ - > BLM	−0.046	0.898	0.369	Not Supported
OTQ - > EEN	0.101	1.849	0.054	Supported
TPR - > BLM	0.046	0.902	0.367	Not Supported
TPR - > EEN	0.242	4.318	0.000	Supported

The p-value test assessed the statistical significance of the relationships [169,173,174], considering p-values between 0.01 and 0.001 as acceptable [169,174]. The analysis reveals that there is a positive correlation between Blended learning motivation (BLM) and Intention to adopt blended learning (IBL) (B = 0.735, p < 0.001), confirming H1. However, the relationship between Educational environment (EEN) and BLM (B = 0.012, p = 0.811) and EEN and IBL (B = −0.038, p = 0.295) did not reach statistical significance, and H2 and H3 were not supported. On the other hand, there is a positive association between Engagement (ENG) and BLM (B = 0.654, p < 0.001), supporting H4. Additionally, ENG negatively influences EEN (B = −0.096, p = 0.027), providing evidence for H5. Furthermore, the relationship between teachers Learning (INL) and EEN (B = 0.247, p < 0.001) is significant, confirming H6. However, INQ did not significantly impact BLM (B = −0.042, p = 0.435), and H7 was not supported. Nevertheless, a positive correlation exists between INQ and EEN (B = 0.134, p = 0.038), validating H8. Moreover, none of the paths from Overall training quality (OTQ) and Teaching presence (TPR) to BLM and EEN reached statistical significance, and H9, H10, H11, and H12 were not supported. Overall, the analysis provides insights into the significant relationships between the constructs in the research model, revealing the factors influencing blended learning motivation and Intention to adopt blended learning among teachers.

## 6 Discussion

Blended learning has emerged as a promising approach in educational contexts, offering a unique combination of online and face-to-face learning experiences. In this study, various factors driving the motivation of teachers toward blended learning were examined. A theoretical motivational model for blended learning was also developed and subjected to empirical testing in Sindh, Pakistan. By reviewing the intentions and attitudes of teachers towards blended learning, this research sought to shed light on the factors that facilitate or hinder the adoption of this innovative training mode. The supported relationship between blended learning motivation and intended blended learning underscores the significance of teachers' motivation in shaping their intention to adopt blended learning. Intrinsically motivated teachers who perceive the benefits of blended learning are likelier to incorporate it into their teaching practice [44,46]. This finding aligns with research emphasizing motivation's role in driving technology adoption intentions. The non-supported relationship between the education environment and blended learning motivation suggests that educators' perceptions of the learning environment may not directly impact their motivation to adopt blended learning. While a positive environment enhances teaching experiences, other factors like teachers' personal beliefs and perceived benefits may be more influential [44]. Similarly, the non-supported relationship between the education environment and intended blended learning indicates that educators' perception of the learning environment may not predict their intention to adopt it. Instead, their beliefs about its efficacy and confidence in using technology might play a more significant role [44].

The supported relationship between teachers' engagement and blended learning motivation emphasizes the positive influence of educators' engagement on their motivation to adopt blended learning. Educators actively engaging with blended learning activities may find it more rewarding and relevant, contributing to higher motivation levels [50,95]. This aligns with research emphasizing the importance of active engagement in fostering motivation. The supported relationship between teachers' engagement and the education environment highlights that active engagement enhances the overall learning context. Engaged teachers contribute to meaningful interactions and collaborative learning experiences, fostering a conducive environment that encourages open communication [138].

The non-supported relationship between teachers' learning and blended learning motivation suggests that teachers' continuous learning and professional development may not directly influence their motivation to adopt blended learning. While ongoing learning enhances teachers' skills, their motivation to adopt blended learning may be influenced by other factors, such as perceived benefits and self-efficacy [44]. Conversely, the supported relationship between teachers’ Learning and the education environment highlights the importance of instructor competence in shaping the learning context. Teachers continually enhancing their knowledge and skills contribute to a more conducive environment, providing participants with opportunities for meaningful interactions and learning experiences [44].

The non-supported relationship between information quality and blended learning motivation suggests that the perceived quality of information in blended learning materials may not directly influence educators' motivation to adopt blended learning. Instead, perceived benefits and self-efficacy may be more prominent in motivating their adoption decisions [46,101]. In contrast, the supported relationship between information quality and the education environment indicates that high-quality content positively contributes to a conducive learning atmosphere. When participants perceive information in online resources as relevant and well-organized, it enhances the learning environment [46,101]. The non-supported relationship between training quality and blended learning motivation suggests that educators' perception of training quality may not significantly influence their motivation to adopt blended learning. While training quality contributes to the overall learning experience of teachers [175]. Conversely, the supported relationship between overall training quality and the education environment highlights the role of training quality in shaping the learning context. Positive perceptions of training quality contribute to a conducive environment, creating opportunities for meaningful interactions and learning experiences [[176], [177], [178]].

The non-supported relationship between teaching presence and blended learning motivation suggests that teachers' presence may not directly influence teachers' motivation to adopt blended learning. At the same time, the teacher's presence enhances interactions [[176], [177], [178]]. In contrast, the supported relationship between teaching presence and the education environment underscores the role of teachers' presence in shaping the learning context. A strong teaching presence fosters interactions, guidance, and feedback, contributing to a positive learning atmosphere [50,95].

The supported relationship between blended learning motivation and intended blended learning underscores the importance of educators' motivation in driving their intention to adopt blended learning. Educators intrinsically motivated to embrace blended learning are likelier to perceive its benefits and value, leading to a stronger intention to adopt it in their teaching practices [44,46]. This result aligns with previous research that emphasizes the role of motivation in predicting technology adoption intentions. The non-supported relationship between the education environment and blended learning motivation suggests that teachers’ perception of the learning environment may not directly influence their motivation to adopt blended learning. While a positive learning environment enhances educators' teaching experiences, their motivation to adopt blended learning may be shaped by other factors, such as personal beliefs and perceived benefits. Similarly, the non-supported relationship between the education environment and intended blended learning indicates that educators' perception of the learning environment may not directly predict their intention to adopt it.

Firstly, teachers’ engagement in blended learning activities showed a strong positive influence on blended learning motivation. This finding aligns with previous research by Refs. [50,138,179], emphasizing the importance of active teachers' engagement in fostering motivation and commitment toward blended learning. Moreover, the study found that teachers learning and professional development positively impact the educational environment. Teachers who continuously enhance their knowledge and skills contribute to a more conducive learning environment, creating opportunities for meaningful interactions and learning experiences for the participants. This result is consistent with the research by Refs. [50,95], which highlighted the significance of teachers' competence in shaping the educational environment in blended learning settings. Another noteworthy finding was the positive relationship between information quality in blended learning materials and the academic environment. When participants perceive the information in the online learning resources as relevant, up-to-date, and well-organized, it contributes to a more supportive and effective educational environment. This finding corroborates the works of [46], who emphasized the importance of high-quality content in promoting a conducive learning atmosphere.

Interestingly, the study found that overall training quality, as perceived by the participants, did not significantly influence blended learning motivation. However, positive perceptions of overall training quality were associated with a slightly more conducive educational environment. This finding suggests that while overall training quality may not directly impact motivation, it still contributes to the participants' overall learning experience and the learning environment. This result aligns with the research by Ref. [132], who emphasized the role of training quality in shaping the participants' learning experiences. Furthermore, teaching presence and active teachers' involvement significantly impacted the educational environment. Instructors providing timely feedback and guidance and encouraging open communication and discussion among participants foster a positive and collaborative learning culture. This finding is consistent with the research by Refs. [50,95], who highlighted the importance of teachers' presence in blended learning environments. The study found that the educational environment does not significantly influence the relationship between the educational environment and blended learning motivation. However, higher levels of teachers’ engagement were associated with a slightly less conducive educational environment. This finding suggests that while the overall educational environment may not directly impact motivation, teachers' engagement plays a crucial role in shaping the learning atmosphere. This result is in line with the work of [138], who emphasized the role of student engagement in influencing the learning environment.

This study contributes to understanding the factors influencing the adoption of blended learning for the professional development of schoolteachers in Sindh, Pakistan. The findings emphasize the importance of teachers' engagement, teachers learning, and information quality in fostering a conducive educational environment. Additionally, the study highlights the significance of teaching presence and active school teachers’ involvement in shaping the learning culture. The results provide valuable insights for education policymakers and administrators in Sindh to design and implement effective blended learning programs that cater to the unique needs and circumstances of teachers and officers in the region. Moreover, this research adds to the existing body of knowledge on blended learning adoption within the context of developing nations, specifically focusing on Pakistan. Nonetheless, this study has some limitations that should be considered for future research.

## 7 Conclusion

This study examined the essential factors affecting the implementation of blended learning for the professional development of teachers in Sindh, Pakistan. Blended learning, combining online and face-to-face learning experiences, holds immense promise for improving educational practices and outcomes, especially in regions like Sindh, where equitable access to quality professional development remains challenging. This research formulated a comprehensive motivational model. The model included factors such as teachers' engagement, learning, information quality, overall training quality, teaching presence, educational environment, blended learning motivation, and the intention to adopt blended learning. We applied SEM analysis for the model analysis by implementing measurement and structural model analysis techniques.

The findings revealed that analyzing the motivating factors significantly influenced the acceptance of blended learning for teachers' professional development—first, intrinsic motivational factors: Several key findings have been discovered in the context of intrinsic motivation. Firstly, Blended Learning Motivation (BLM) has surfaced as a compelling driver, significantly influencing teachers' intention to adopt blended learning. This intrinsic motivation underscores the importance of teachers' interest and engagement in the blended learning process. It signifies that when teachers are genuinely motivated and enthusiastic about blended learning, they are more likely to embrace it as an effective avenue for their professional development. Moreover, Engagement (ENG), characterized by active participation and interaction within the blended learning environment, has emerged as a prominent intrinsic motivator. The study affirmed the pivotal role of engaging learning experiences in motivating teachers toward adopting blended learning. When teachers actively engage with the learning materials, their peers, and students, they are more inclined to perceive the benefits of blended learning, thus increasing their motivation to adopt it.

Extrinsic motivational factors: The study revealed several significant findings on the extrinsic side of motivation. Teachers' learning, representing an extrinsic motivator, while not statistically significant in this context, still holds significance. Continuous professional development of teachers remains required as it contributes to creating a conducive learning environment in schools and at the classroom level. Teachers who actively enhance their knowledge and skills can positively impact the quality of education and the overall learning experience. Similarly, Information quality, another extrinsic factor, did not directly impact blended learning motivation in this study. However, providing high-quality content remains crucial in shaping the educational environment. When teachers perceive the information in blended learning materials as relevant, up-to-date, and well-organized, it contributes to a more supportive and effective educational environment. This study has highlighted the intricate relationships between intrinsic and extrinsic motivators in influencing teachers' acceptance of blended learning for professional development. Intrinsic motivation, rooted in personal interest and engagement, emerges as a potent force. Simultaneously, while not significantly significant, extrinsic factors such as teachers' learning and information quality engage in pivotal roles in creating a conducive learning atmosphere. These findings provide valuable insights for educational policymakers and practitioners seeking to enhance the implementation of blended learning initiatives in the context of teachers' professional development. This study's findings are highly relevant for educational policymakers and practitioners in Sindh, Pakistan. It underscores the significance of nurturing teachers' motivation to embrace blended learning and emphasizes creating engaging and compelling learning experiences. Additionally, enhancing the quality of training content, providing robust teacher support, and improving overall training quality can significantly contribute to shaping a positive educational environment facilitating the widespread adoption of blended learning in government schools.

### 7.1 Limitations

Despite the valuable insights obtained from this study, it is essential to acknowledge its limitations. First, the research was limited to government schoolteachers in Sindh, Pakistan, and may not fully represent other educational contexts or diverse groups of teachers. The findings should be interpreted with caution when generalizing them to different populations. Second, the study relied on self-reported data, which might be subject to response biases and social desirability effects. Another limitation is the focus on teacher perspectives, neglecting the views of students and administrators, who are essential stakeholders in adopting blended learning. Future research could include a more comprehensive examination by incorporating multiple stakeholders' perspectives.

### 7.2 Implications and recommendations

#### 7.2.1 Theoretical implications

The findings of this study contribute to the existing literature on blended learning (BL) adoption by highlighting the motivational factors that influence teachers' intentions to adopt BL. The positive relationship between BL motivation and the intention to adopt BL emphasizes the importance of adopting intrinsic motivation among educators. This study also reveals the significance of teachers' engagement, learning, and teaching presence in shaping the educational environment and motivation for BL. The theoretical framework developed in this research can serve as a foundation for future studies exploring the adoption of BL in various educational contexts.

Furthermore, the non-significant relationships between certain constructs suggest that additional factors might influence BL adoption. Future research should investigate contextual factors, such as cultural influences, technological infrastructure, and institutional policies, to provide a more comprehensive understanding of the determinants of BL adoption. Exploring the perspectives of students and administrators can also offer valuable insights into the multifaceted nature of BL implementation and its impact on educational outcomes.

#### 7.2.2 Practical implications and recommendations

The practical implications of this study are significant for educational policymakers, administrators, teachers, and other stakeholders involved in implementing BL. The positive impact of BL motivation on the intention to adopt BL underscores the need for strategies that enhance teachers' motivation and enthusiasm for BL.

For educational policymakers, it is essential to develop robust policies supporting BL integration in educational institutions. This includes establishing clear guidelines and policies addressing infrastructure, resources, and training programs. Allocating funding for BL initiatives is important to ensure that schools and teachers have access to the necessary technological tools and resources. Furthermore, promoting professional development programs focused on BL can help teachers effectively acquire the skills and knowledge required to integrate BL into their teaching practices.

School administrators should focus on providing regular training sessions and workshops for teachers to enhance their understanding and application of BL. Ensuring ongoing support through mentoring and collaborative learning communities can significantly impact the successful adoption of BL. Investing in and maintaining technological infrastructure that supports BL, including high-speed internet, digital devices, and learning management systems, is also vital. Additionally, fostering a positive and collaborative educational environment encourages innovation and experimentation with BL methodologies, recognizing and rewarding teachers' efforts and achievements in implementing BL. Teachers also play an essential role in successfully adopting BL and should actively engage in continuous learning by participating in professional development opportunities and staying updated with the latest trends and best practices in BL. Collaborating with peers to share insights, strategies, and resources for BL can lead to developing of innovative and effective BL practices. Focusing on student-centered learning by designing BL activities that are engaging and relevant to student's needs and interests and incorporating interactive and collaborative elements can foster student engagement and motivation.

Furthermore, Students should also actively participate in BL activities, taking advantage of the opportunities for personalized learning and interaction with peers and instructors. Providing feedback to teachers and administrators can help improve the quality and effectiveness of BL programs, ensuring that student needs and preferences are considered. Research scholars should continue to investigate additional factors, such as cultural influences and technological infrastructure, that may impact the adoption of BL. Including the perspectives of students, administrators, and other stakeholders in research studies can provide a comprehensive understanding of the factors influencing BL adoption, ultimately enhancing the quality of teaching and learning in the digital age. By implementing these recommendations, stakeholders can create an environment that supports the successful adoption of BL, leading to positive outcomes for teachers' professional development and the overall educational experience. The findings of this study provide valuable results for educational policymakers and practitioners in Sindh, Pakistan, and beyond, offering practical guidance for the effective integration of BL in educational settings.

### 7.3 Future work

Future research could expand its scope to explore additional factors that might influence the adoption of blended learning. Investigating the impact of institutional support, technological infrastructure, and contextual factors can provide a more comprehensive understanding of the challenges and opportunities for blended learning implementation. Additionally, longitudinal studies could track teachers' experiences over time to assess the long-term effects of blended learning adoption on their teaching practices and student outcomes. Furthermore, the study could be replicated in other regions of Pakistan and different countries to compare results and identify potential cross-cultural differences in the factors influencing blended learning adoption. Exploring the role of innovative technologies, such as artificial intelligence and virtual reality, in enhancing blended learning experiences could be another avenue for future research. In conclusion, while this study contributes valuable insights to blended learning adoption in Sindh, Pakistan, further research is warranted to address the identified limitations and advance our understanding of the complex interplay of factors shaping teachers' intentions to adopt blended learning.

## Funding support

This work was funded by the Researchers Supporting Project Number (RSPD2024R564) 10.13039/501100002383King Saud University, Riyadh, Saudi Arabia.

## Informed consent statement

All participants provided informed consent before joining the study.

## Data availability statement

Data will be made available on request to corresponding authors.

## Ethical approval statement

The research study mentioned above involved the collection of data from UTM Malaysia, and prior ethical approval was duly obtained, under Reference No. UTM.J.13.01/13.14/1/88 Jld.23 (75)/Dated: 1-06-2023 and under RMC research project no. Q. J130000.21A2.07E10.

## Ethical statement

In accordance with ethical standards, I hereby confirm that the research study mentioned above involved the collection of data from school teachers, and prior ethical approval was duly obtained from the Directorate of School Education, Shaheed Benazirabad Region, Government of Sindh, under Reference No. No.DSE (ES&HS) A. D(F.A)/SBA/17853/NShah/Dated: 16-07-2023. Additionally, formal permissions were granted by the University of Shaheed Benazir Bhutto University via Letter No. SBBU/Edu/120/Dated: 13-4-2023 and the University Teknologi Malaysia (UTM) through Letter Reference No. UTM.J.13.01/13.14/1/88 Jld.23(75)/Dated: 1-06-2023 and under RMC research project No. Q. J130000.21A2.07E10. Copies of these approval letters are attached herewith for reference and verification, confirming that all necessary ethical and regulatory requirements have been met throughout the course of this research project.

## CRediT authorship contribution statement

**Nisar Ahmed Dahri:** Writing – review & editing, Writing – original draft, Validation. **Noraffandy Yahaya:** Project administration, Formal analysis, Conceptualization. **Waleed Mugahed Al-Rahmi:** Resources, Methodology, Funding acquisition, Conceptualization. **Haitham Ameen Noman:** Writing – review & editing, Supervision. **Fahad Alblehai:** Visualization, Supervision, Software. **Yusri Bin Kamin:** Supervision, Software, Methodology, Conceptualization. **Rahim Bux Soomro:** Methodology, Formal analysis. **Anna Shutaleva:** Writing – review & editing, Conceptualization. **Ahmad Samed Al-Adwan:** Funding acquisition, Investigation, Resources, Validation.

## Declaration of competing interest

The authors declare that they have no known competing financial interests or personal relationships that could have appeared to influence the work reported in this paper.
