# Supplementary material for: Investigating the motivating factors that influence the adoption of blended learning for teachers’ professional development
Source: Heliyon. 2024 Jul 20;10(15):e34900. doi: 10.1016/j.heliyon.2024.e34900 (PMC11320316; doi:10.1016/j.heliyon.2024.e34900)
Supplement: Multimedia component 1 [file mmc1.docx]

**Questionnaire**

Investigating the Motivating Factors That Influence the Adoption of Blended Learning for Teachers’ Professional Development

**Section A: Demographic Information-** Tells us about yourself.

| Name (Optional) | |  | | | |
| --- | --- | --- | --- | --- | --- |
| Age group | 20-30 (1) | 31-40 (2) | 41-50 (3) | 51-60 | Other (5) |
| i) Gender of the respondent | | Male 🞐 Female 🞐 | | | |
| ii) Qualification (Academic & Professional) | | Bachelor 🞐 Masters 🞐 Ph.D. 🞐 | | | |
| iii) Teaching Experience | | ___________________________________________ | | | |
| iv) Department/ Type of School | | Public 🞐 Private 🞐 | | | |
| v) Geographical Location | | Urban 🞐 Rural 🞐 | | | |
| vi) Training Experience: Have you previously participated in any online or blended learning training programs? | | Yes 🞐 No 🞐 | | | |
| vii) Access to Technology: Do you have regular access to a computer or a smartphone with internet connectivity? | | Yes 🞐 No 🞐 | | | |
| viii) Preferred Mode of Learning: In general, do you prefer face-to-face, online, or blended learning for professional development? | | Face to face 🞐 Online 🞐 Blended 🞐 | | | |
| ix)Personal Challenges in Training Attendance: Are there any personal challenges or constraints that hinder your participation in face-to-face trainings? | | Lack of time 🞐 Lack of resources 🞐 None 🞐 Other 🞐 | | | |
| x)Availability for Training: How willing are you to attend a blended learning training that combines online and face-to-face sessions? | | Very Willing 🞐 Somewhat willing 🞐 Neutral 🞐 Somewhat unwilling 🞐 Very Unwilling 🞐 | | | |

**Section B:** AI Adoption Tool

*Please rate the extent to which you agree with each statement below.*

(Please check √ the most appropriate option for each statement below)

1=Strongly Disagree (SD) 2= Disagree (D) 3= Neutral(N) 4= Agree(A) 5= Strongly agree (SA)

| **Students' Engagement (ENG1-ENG4)** | |  |  |  |  |  |
| --- | --- | --- | --- | --- | --- | --- |
| ENG1 | I was motivated to actively participate in the blended learning training. |  |  |  |  |  |
| ENG2 | The training content and activities captured my interest and attention. |  |  |  |  |  |
| ENG3 | I felt engaged and involved in the learning process throughout the training. |  |  |  |  |  |
| ENG4 | Overall, I found the training experience enjoyable and fulfilling. |  |  |  |  |  |
| **Instructor Learning (INL1-INL4)** | |  |  |  |  |  |
| INL1 | The instructors who facilitated the blended learning training were knowledgeable and competent. |  |  |  |  |  |
| INL2 | The instructors were responsive to my questions and concerns during the training. |  |  |  |  |  |
| INL3 | The instructors effectively explained complex concepts and clarified doubts. |  |  |  |  |  |
| INL4 | I found the instructors' teaching methods to be effective and engaging. |  |  |  |  |  |
| **Information Quality (INQ1-INQ4)** | |  |  |  |  |  |
| INQ1 | The information provided in the online learning materials was relevant and up to date. |  |  |  |  |  |
| INQ2 | I found it easy to access and understand the training resources and materials. |  |  |  |  |  |
| INQ3 | The training content was well-organized and logically presented. |  |  |  |  |  |
| INQ4 | The information provided was helpful in achieving the learning objectives. |  |  |  |  |  |
| **Training Quality (OTQ1-OTQ5)** | |  |  |  |  |  |
| OTQ1 | I would rate the overall quality of the blended learning training as high. |  |  |  |  |  |
| OTQ2 | The training objectives and outcomes were clearly communicated at the beginning. |  |  |  |  |  |
| OTQ3 | The training met my expectations and learning needs. |  |  |  |  |  |
| OTQ4 | I found the blended learning approach to be effective for my professional development. |  |  |  |  |  |
| OTQ5 | I would recommend this training to my colleagues and peers. |  |  |  |  |  |
| **Teaching Presence (TPR1-TPR4)** | |  |  |  |  |  |
| TPR1 | The instructors provided timely feedback and support during the online learning phase. |  |  |  |  |  |
| TPR2 | The instructors actively guided and facilitated face-to-face sessions. |  |  |  |  |  |
| TPR3 | The instructors encouraged open communication and discussion among participants. |  |  |  |  |  |
| TPR4 | The instructors fostered a positive and inclusive learning environment. |  |  |  |  |  |
| **Educational Environment (EEN1-EEN3)** | |  |  |  |  |  |
| EEN1 | The overall learning environment was conducive to my learning experience. |  |  |  |  |  |
| EEN2 | The training resources and facilities were accessible and user-friendly. |  |  |  |  |  |
| EEN3 | The educational environment fostered a positive and collaborative learning culture. |  |  |  |  |  |
| **Blended Learning Motivation (BLN1-BLM3)** | |  |  |  |  |  |
| BLM1 | I was motivated to actively engage in the blended learning training. |  |  |  |  |  |
| BLM2 | The blended learning approach enhanced my motivation to learn. |  |  |  |  |  |
| BLM3 | I believe that blended learning is an effective way to enhance professional development. |  |  |  |  |  |
| **Intention to Adopt Blended Learning** | |  |  |  |  |  |
| IBL1 | I intend to adopt blended learning for future professional development. |  |  |  |  |  |
| IBL2 | I am likely to recommend blended learning to my colleagues for their trainings. |  |  |  |  |  |
| IBL3 | I plan to incorporate blended learning strategies in my teaching practices. |  |  |  |  |  |
